# Cerebral Ischemia Protection After Aneurysmal Subarachnoid Hemorrhage: CSF Nimodipine Levels After Intravenous Versus Oral Nimodipine Administration

**DOI:** 10.1002/cpt.3499

**Published:** 2024-11-19

**Authors:** Miriam M. Moser, Karl Rössler, Dorian Hirschmann, Leon Gramss, Ammar Tahir, Walter Plöchl, Johannes Herta, Andrea Reinprecht, Markus Zeitlinger, Arthur Hosmann

**Affiliations:** ^1^ Department of Neurosurgery Medical University of Vienna Vienna Austria; ^2^ Department of Pharmaceutical Sciences University of Vienna Vienna Austria; ^3^ Department of Anaesthesia, Intensive Care Medicine and Pain Medicine, Division of General Anaesthesia and Intensive Care Medicine Medical University of Vienna Vienna Austria; ^4^ Department of Clinical Pharmacology Medical University of Vienna Vienna Austria

## Abstract

There is accumulating evidence that cerebrospinal fluid (CSF) concentrations of nimodipine correlate with long‐term outcome of patients after subarachnoidal hemorrhage (aSAH) by impeding cerebral ischemia. However, pharmacological data on simultaneous serum vs. CSF and intraparenchymal nimodipine values are rarely reported in larger patient groups. Nimodipine concentrations were determined in plasma, CSF, and cerebral interstitial fluid (ISF), at steady state after oral (6 × 60 mg/day) and intravenous (0.5, 1, 1.5 and 2 mg/h) administrations in 10 patients after aSAH. Area under the concentration time curve (AUC_0–24_) for intravenous nimodipine was highest at an infusion rate of 2 mg/h in plasma (1335.87 ± 591.09 mg*h/L), followed by CSF (39.53 ± 23.07 mg*h/L), resulting in an overall CSF penetration ratio of 3.8% (±1.5) (AUC_CSF_/AUC_plasma_). In contrast, nimodipine levels were significantly lower in both plasma (AUC_0–24_ 298.32 ± 206.52 mg*h/L) and CSF (AUC_0–24_ 34.8 ± 16.56 mg*h/L) after oral administration. In cerebral ISF, low amounts of nimodipine were detectable in only 4 patients at an infusion rate of 1.5 and 2 mg/h as well as following oral administration. We found significantly higher CSF nimodipine levels in patients during intravenous compared to oral administration. In contrast, only low amounts of nimodipine were detected in the ISF after both oral and intravenous administration. Our findings strongly suggest that the main clinical nimodipine effect of impeding life threatening cerebral ischemia is mediated through significant higher CSF levels after intravenous administration, more likely effective than oral administration.

**Study Highlights**

**WHAT IS THE CURRENT KNOWLEDGE ON THE TOPIC?**

Aneurysmal subarachnoid hemorrhage (aSAH) can result in poor functional outcome. A significant complication after aSAH is delayed cerebral ischemia (DCI). Besides surgical or interventional treatment of the ruptured aneurysm, guidelines recommend the calcium channel blocker nimodipine for the prevention of delayed cerebral ischemia.

The administration of 60 mg of nimodipine orally every 4 hours has shown beneficial effects on outcome of patients, but data on intravenous administration are limited.

**WHAT QUESTION DID THIS STUDY ADDRESS?**

In this study, we prospectively investigated the pharmacokinetics of 60 mg of oral and 0.5, 1, 1.5, and 2 mg/h intravenous nimodipine in plasma, cerebrospinal fluid, and interstitial cerebrospinal fluid in 10 patients after aneurysmal subarachnoid hemorrhage.

**WHAT DOES THIS STUDY ADD TO OUR KNOWLEDGE?**

According to our data, significantly higher concentrations of nimodipine can be achieved in both plasma and CSF during intravenous compared to oral administration. Conversely, in cerebral interstitial fluid, only low amounts of nimodipine are found after both routes, with detection limited to less than half of the patients. These findings strongly suggest that nimodipine primarily exerts its beneficial effect on functional outcome by impeding cerebral ischemia through vasodilation via the blood–CSF barrier rather than neuroprotection via the blood–brain barrier.

**HOW MIGHT THIS CHANGE CLINICAL PHARMACOLOGY OR TRANSLATIONAL SCIENCE?**

At present, clinical guidelines recommend the oral administration of 60 mg of nimodipine. Our findings suggest that intravenous administration achieves higher concentrations in both plasma and CSF, which may lead to greater clinical effectiveness.

Aneurysmal subarachnoid hemorrhage (aSAH) can result in poor functional outcome.^1^ A significant complication post‐aSAH is delayed cerebral ischemia (DCI), which was initially attributed primarily to cerebral vasospasm, but nowadays is seen as a combination of vasospasm and other processes including breakdown of blood–brain barrier, impairment of autoregulation, and neuroinflammatory processes.^1^, ^2^, ^3^

Besides surgical or interventional treatment of the ruptured aneurysm, guidelines recommend the calcium channel blocker nimodipine for the prevention of delayed cerebral ischemia.^2^

Former studies^4^, ^5^, ^6^, ^7^, ^8^, ^9^ showed a beneficial effect on outcome of patients receiving 60 mg of nimodipine orally every four hours, but data on intravenous administration are limited.^2^, ^10^ However, there is accumulating evidence that cerebrospinal fluid (CSF) concentrations of nimodipine correlate with long‐term outcome of patients after aSAH.^11^ Despite its established efficacy, the precise mode of action remains unknown. The observed benefits are hypothesized to be due to its vasodilative properties on cerebrovasculature or neuroprotective effects by reducing intracellular calcium and preventing cellular apoptosis.^12^

Nevertheless, besides its positive impact on outcome after aSAH, nimodipine may also induce systemic hypotension and worsen cerebral perfusion.^3^ A prior study conducted by our group revealed a significant reduction of cerebral perfusion pressure and brain tissue oxygen tension following oral nimodipine administration, but without discernible effects on cerebral metabolism.^13^

Intravenous nimodipine infusion has the potential to amplify these side effects. Simultaneously, it may concurrently elevate cerebral concentrations, thereby possibly enhancing its vasodilative and potential neuroprotective effects. P‐glycoprotein (P‐gp) is known to restrict the permeation of nimodipine into the brain,^14^, ^15^ so currently, it is unclear whether nimodipine can permeate the blood–brain barrier in humans at all.

Therefore, this study was set up to investigate the ability of nimodipine to penetrate into the brain following oral and intravenous administration in patients after aSAH and determine its pharmacokinetics in plasma, CSF, and cerebral interstitial fluid (ISF).

## METHODS

### Population

This prospective study included 10 patients between 11/2020 and 10/2022 with severe aSAH requiring deep sedation and multimodal neuromonitoring and cerebral microdialysis. Ten patients are assumed to be sufficient to allow for significant pharmacokinetic description.

The study drug was investigated at oral and intra‐venous administrations in the same patients, as switch from intra‐venous to oral administration was routinely performed after 10–14 days.

The study protocol was approved by the ethics committee of the Medical University of Vienna (EK‐Nr. 1774/2020, EudraCT 2020‐002968‐31) and the study was conducted at the neurosurgical intensive care unit of the Medical University of Vienna. All study procedures adhered to the principles of the Declaration of Helsinki.

Patients meeting study criteria were initially unable to provide written consent due to sedation and mechanical ventilation. Upon regaining consciousness, patients were informed about the study, and retrospective permission was obtained. Follow‐up of outcome was evaluated after 3 months (modified Rankin Scale (mRS)).

### Cerebral microdialysis

A Bolt Microdialysis Catheter (M Dialysis AB, Stockholm, Sweden), perfused with artificial CSF (Perfusion Fluid CNS, M Dialysis AB, Stockholm, Sweden) at a flow rate of 0.3 μL/min (107 Microdialysis Pump, M Dialysis AB, Stockholm, Sweden), was placed side by side with a NEUROVENT‐PTO 2L catheter (Raumedic AG, Helmbrechts, Germany) in the frontal lobe on the side of the ruptured aneurysm or at the side of maximal extension of subarachnoid blood, respectively.

### Study medication

Nimodipine was administered as a routine measure in all included patients. Intravenous administration was initiated at a dose of 0.5 mg/h, and the dose was incrementally increased in 0.5 mg/h steps until the maximum dose of 2 mg/h was reached. All measurements were performed at steady state, meaning that the dose was changed at least 12 hours prior to pharmacokinetic (PK) measurement.

### Sampling

Delivery and storage of nimodipine were according to standard regulations of the pharmacy of the general hospital of Vienna. Nimodipine concentrations in plasma, CSF, and interstitial fluid were measured at steady state of 0.5, 1, 1.5, and 2 mg/h and 60 mg oral administration. Cerebral microdialysis samples were collected every 2 hours. In between, microvials were collected to measure cerebral metabolites bedside. Concomitantly with microdialysis vial collection, blood and cerebrospinal fluid samples were collected.

Approximately 5 mL of blood was drawn from an arterial catheter for plasma measurement. CSF was collected from an external ventricular drainage. The first milliliter of CSF was discarded and the second milliliter was stored for further analysis. All samples were protected from light and kept on ice, immediately centrifuged, and stored at −80°C thereafter. To determine the individual in vivo probe recovery for nimodipine, retrodialysis was conducted. In each patient included in the study, the microdialysis probe was perfused with a solution containing 50 μg/mL nimodipine at a flow rate of 0.3 μg/mL (*C*_in_). Two microdialysis samples (after an equilibration time of one hour) were collected for each patient to determine the average nimodipine concentration (*C*_out_). The recovery was computed as the ratio of drug lost during passage (*C*_in_–*C*_out_) and entering the microdialysis probe.

### Forward‐ and retrodialysis of nimodipine in vitro

In vitro microdialysis was performed to imitate recovery rates under controlled conditions and to test whether recovery is equal in both directions (forward/reverse). The concentrations were based on the concentration range measured *in vivo*. Three microdialysis probes were placed separately in glass vials containing 0.0001 pg/μL of nimodipine solution and were constantly perfused with 0.9% saline at a flow rate of 0.3 μL/min. After 3 hours of sampling, the probes were placed in the next higher concentrations (0.01 and 1 pg/μL), samples were collected over 180 minutes. Reverse dialysis concentrations in the perfusion solution of the microdialysis pump were determined as 0.1, 1, and 50,000 pg/μL. Last concentration was set similar to the retrodialysis concentration performed in vivo. The respective nimodipine solution was used as perfusion solution and probes were perfused at a flow rate of 0.3 μL/min, and 0.9% saline was applied as immersion solution for all three probes in separate glass vials. Sampling timepoints were similar to forward dialysis.

### Drug assay

Nimodipine (>99% Purity, Sigma Aldrich – Merck) and Nimodipine‐d7 (internal standard, IS, >99% Purity, Cayman Chemicals) were separated using an isocratic elution using an UHPLC ExionLC AD System (Joint venture Shimadzu and AB Sciex – Germany) equipped with a reversed‐phase C18 column (Luna Omega; 2.1 mm × 5 cm, 1.6 μm, C18 100 Å, Phenomenex – Germany) with the column temperature maintained at 40°C. The isocratic elution was performed using a quaternary mobile phase composed of equal parts of water, acetonitrile, methanol, 2‐propanol, and 0.1% (v/v) of formic acid. The total run time of the sample was 3 minutes. The flow rate was set 350 μL/min. The autosampler temperature was kept at 4°C. Mass spectrometric detection was performed using turbo ion source ESI Qtrap 4,500 mass spectrometer (AB Sciex – Germany) multiple reaction monitoring (MRM) mode (581 cycles with 0.31 seconds per cycle). Data acquisition and analysis were performed using the vendor software Analyst v.1.7.3.

### Pharmacokinetics

The following results describe the total concentration of nimodipine, including both bound and unbound drug. Based on existing literature,^16^ nimodipine has a high degree of plasma protein binding of about 98%; this must be considered when interpreting the presented results of this study. Moreover, the plasma half‐life of intravenous and oral nimodipine is known to be between 0.9 and 1.5 hours for intravenous administration and between 1.7 and 7.2 hours for oral administration,^3^ which was used for further calculations.

The area under the curve at steady‐state conditions (AUC) was calculated for each dose of nimodipine (i.e. 0.5, 1, 1.5, 2 mg/h, and 60 mg p.o.). The AUC was calculated over 7 hours and multiplied by a factor of 3.42857 (24 divided by 7) for intravenous and 6 (24 divided by 4) for oral administration to obtain the AUC for 24 hours.

${\mathrm{AUC}}_{0-\infty }$ was calculated as ${\mathrm{AUC}}_{0-\infty }=\frac{\text{Concentratio}n_{\text{mean}}}{k_{\mathrm{el}}}$.

To evaluate the drug penetration of nimodipine, the ratio between the AUC in CSF and plasma (AUC_CSF_/AUC_plasma_) was calculated.

The relative bioavailability in plasma and CSF of oral compared to intravenous administration of nimodipine was calculated as $F=\frac{\mathrm{AU}C_{p.o.\;0-24h}*\mathrm{Dos}e_{i.v.\;0-24h}}{\mathrm{AU}C_{i.v.\;0-24h}*\mathrm{Dos}e_{p.o.\;0-24h}}*100$.

The total clearance was calculated as ${\mathrm{Cl}}_{\text{total}}=\frac{\mathrm{Dos}e_{i.v.}}{{\mathrm{AUC}}_{0-\infty }}$

### Statistical analysis

Statistical analysis was conducted using SPSS statistics 29 (IBM Corp., Armonk, NY), MS Excel für Mac Version 16.75.2 and Prism 9 for macOS. Single missing values were reported and skipped for PK analysis as PK measurements were performed under steady‐state conditions.

Descriptive statistics are presented as mean ± standard deviation for plasma and CSF and as median and range for the cerebral ISF data. Figures were created using Prism 9 for macOS. Correlation of concentration between compartments was calculated with Pearson Correlation coefficient and results were defined as significant at a two‐sided significance level < 0.05. Compartmental differences between intravenous and oral administration and differences of different doses were calculated using paired samples t‐test or Mann–Whitney U test for unequally distributed data.

### Ethics statement

The study protocol was approved by the ethics committee of the Medical University of Vienna (EK‐Nr. 1774/2020, EudraCT 2020‐002968‐31).

## RESULTS

### Population

In this prospective study, 10 patients suffering from severe aSAH (Hunt&Hess grade 4 ± 1), requiring deep sedation and cerebral microdialysis were included. The patients' and sampling characteristics are presented in detail in **Table** **1**.

**Table 1:** Patient demographics

Patient characteristics	
Patients included	10
Age (years)	55 ± 10 (range 36–68)
Sex	
Female	6
Male	4
Hunt & Hess	4 ± 1
Average BMI	23 ± 3
Surgical intervention	
Clipping	5
Coiling	5
Start of multimodality monitoring after bleeding event (days)	1 ± 1
Mean duration of multimodality monitoring (days)	16 ± 3
PK analysis after aSAH (days)	
0.5 mg/h i.v.	5 (±3)
1 mg/h i.v.	6 (±2)
1.5 mg/h i.v.	8 (±5)
2 mg/h i.v.	9 (±2)
60 mg p.o.	16 (±3)
Outcome after 3 months	
mRS	3 (range 0–5)

aSAH, aneurysmal subarachnoid hemorrhage; BMI, body mass index; mRS, modified Rankin Scale.

Total nimodipine concentrations (bound and unbound) were measured at steady state after intravenous administration in 10 patients. In one patient, the microdialysis probe was removed before transitioning to oral nimodipine administration, resulting in the inclusion of only nine patients in the oral PK analysis.

At times CSF sampling could not be obtained due to slit ventricles, so the sample was omitted. In such instances, analysis was limited to the remaining samples collected of the day. In one patient, the entire period of 0.5 mg/h for CSF PK was missing, and only nine patients were included in this analysis. Cerebral ISF data for 1.5 mg/h were missing in one patient due to a microdialysis probe malfunction.

### *In vivo* and *in vitro* retrodialysis

In vivo, the individual relative probe recovery was 99% (±0). Concomitantly, in vitro retrodialysis tests showed a reproducible recovery rate of 99% (±0) with a nimodipine concentration in perfusion fluid of 50,000 pg/μL, equivalent to the in vivo conditions. However, the recovery rate was highly variable in the in vitro experiments. The inconsistency indicates that the drug adheres to the membrane and is only partially permeable, which precludes the interpretation of the results as absolute values. Consequently, no correction for recovery rate was performed in the in vivo results and the concentrations only indicate whether or not nimodipine reaches the cerebral ISF.

### Pharmacokinetics

The relative bioavailability in plasma of oral nimodipine compared to intravenous administration ranged between 1.7% and 3%, the relative bioavailability in CSF of oral nimodipine compared to intravenous administration ranged between 4.7% and 11.7% (**Table** **2**). The total clearance of intravenous nimodipine was 0.019–0.033 at 0.5–2 mg/h continuous intravenous infusion, respectively (Cl_total_ of 0.5 mg/h = 0.019, of 1 mg/h = 0.024, of 1.5 mg/h = 0.026, of 2 mg/h = 0.033).

**Table 2:** Relative bioavailability

Relative bioavailability of nimodipine		
Dose in 24 hours	Relative plasma bioavailability (%)	Relative CSF bioavailability (%)
360 mg p.o./12 mg i.v. (0.5 mg/h)	1.7	4.7
360 mg p.o./24 mg i.v. (1 mg/h)	2.1	6.9
360 mg p.o./36 mg i.v. (1.5 mg/h)	2.3	10.6
360 mg p.o./48 mg i.v. (2 mg/h)	3	11.7

CSF, cerebrospinal fluid.

#### Plasma

**Figure 1:**
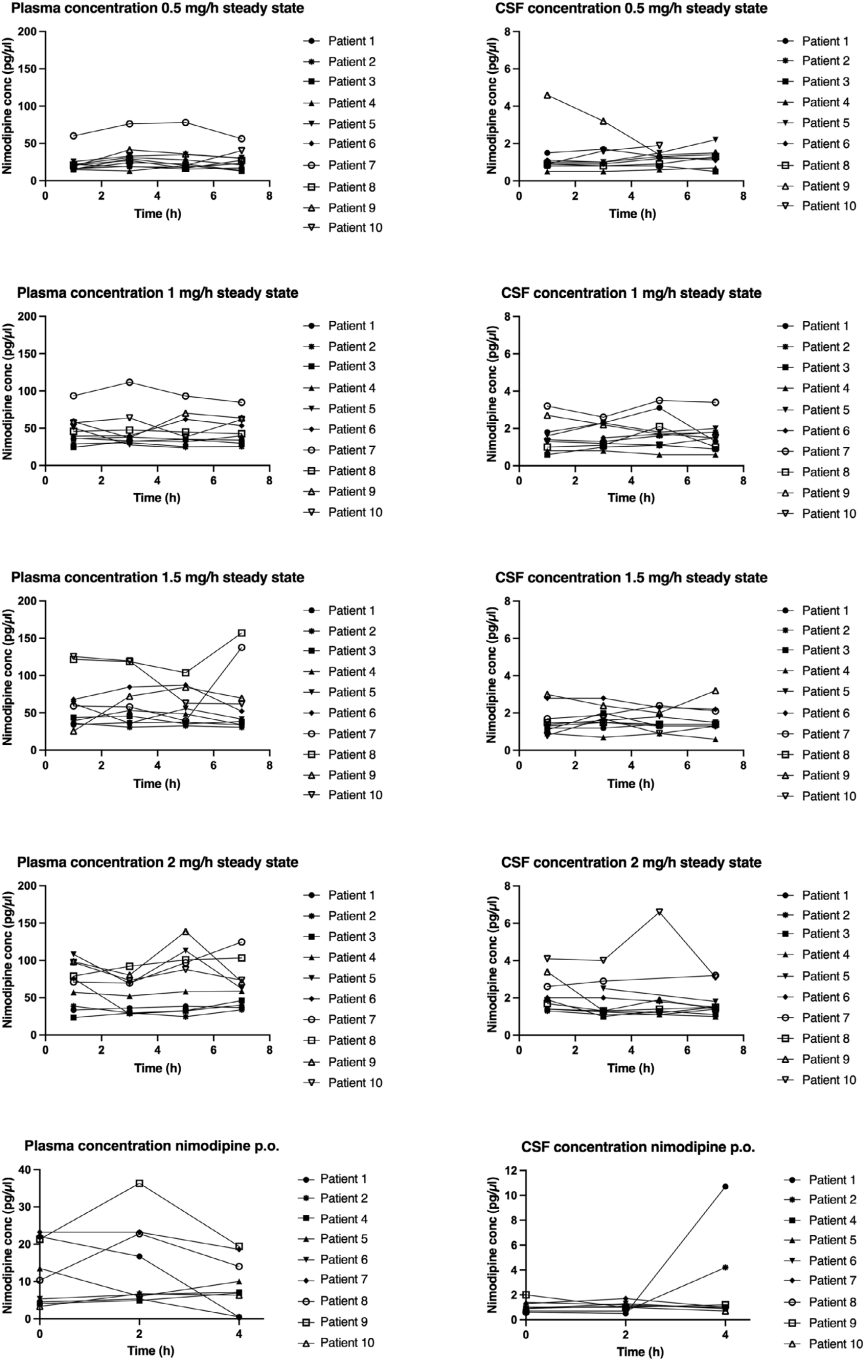
Nimodipine in plasma and cerebrospinal fluid. Variability of nimodipine concentration in plasma and cerebrospinal fluid (CSF) at steady state in 10 patients at 0.5, 1, 1.5, and 2 mg/h over 7 hours at 4 different timepoints and at 60 mg oral dose over 4 hours at 3 different time points.

**Figure 2:**
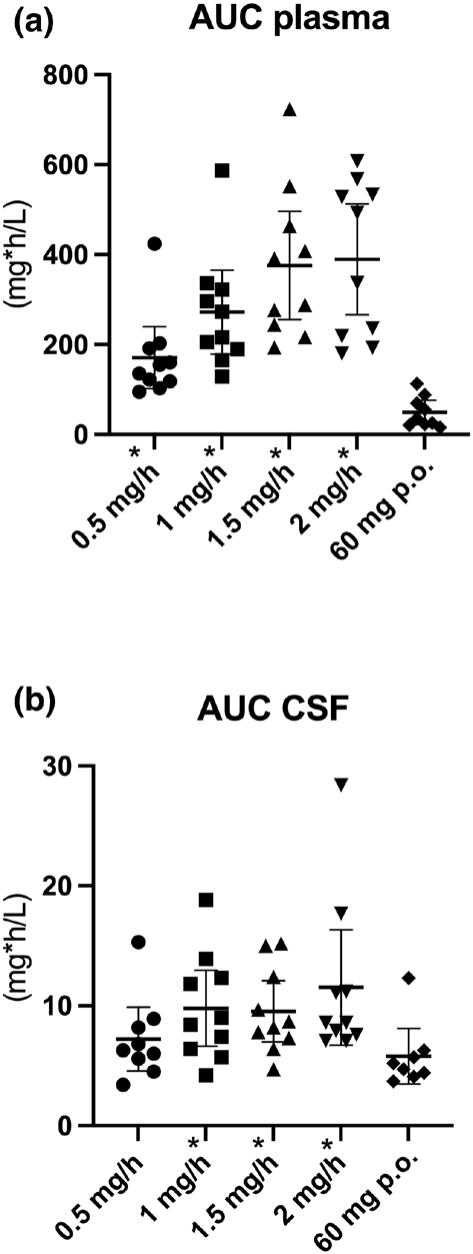
Dot plots showing different nimodipine concentrations according to dose and route of administration. (**a**) Dot plots show individual data points and the median and 95% confidence interval (CI) of AUC_0–7_ in plasma at 0.5, 1, 1.5, and 2 mg/h continuous nimodipine infusion and AUC_0–4_ at oral administration of 60 mg of nimodipine every 4 hours. (**b**) Dot plots show individual data points and the median and 95% CI of nimodipine AUC_0–7_ in CSF at 0.5, 1, 1.5, and 2 mg/h continuous nimodipine infusion and AUC_0–4_ at oral administration of 60 mg of nimodipine every 4 h. AUC, area under the curve; CSF, cerebrospinal fluid, *statistically significant difference between oral and corresponding intravenous dose of nimodipine.

For intravenous administration, the mean concentrations of nimodipine in plasma and AUC_0–24_ are presented in detail in **Table** **3** and **Figure** **1**. Mean AUC_0–24_ in plasma was 585.91 (± 329.28) mg*h/L at 0.5 mg/h (12 mg/day), 932.57 (± 445.70) mg*h/L at 1 mg/h (24 mg/day), 1289.04 (± 576.65) mg*h/L at 1.5 mg/h (36 mg/day), and 1335.87 (±591.09) mg*h/L at 2 mg/h (48 mg/day) (**Figure** **2**). Thus, the increase in plasma concentration was dose‐dependent. A significant increase in absolute plasma concentration was observed between 0.5 mg/h and 1–2 mg/h (*P* < 0.05, between 0.5 and 1 mg/h *P* < 0.001, between 1 and 1.5 mg/h *P* = 0.09, between 1.5 and 2 mg/h *P* = 0.75, between 0.5 and 1 mg/h *P* < 0.001, between 0.5 and 1.5 mg/h *P* = 0.004, between 0.5 and 2 mg/h *P* < 0.001, between 1 and 1.5 mg/h *P* = 0.09, and between 1 and 2 mg/h *P* = 0.03; absolute values in **Table** **3**). The increase was not significant between 1 mg/h and 1.5–2 mg/h and between 1.5 mg/h and 2 mg/h (*P* > 0.05, between 1 and 1.5 mg/h *P* = 0.09, between 1.5 and 2 mg/h *P* = 0.75; absolute values in **Table** **3**).

**Table 3:** Nimodipine concentration in plasma, cerebrospinal fluid, and cerebral interstitial fluid at intravenous and oral administration

	Plasma	CSF	Cerebral ISF
**Continuous intravenous infusion of nimodipine (steady state)**			
Mean concentration of nimodipine ± SD (pg/μL)			Median (range)
0.5 mg/h	27.64 ± 15.07	1.28 ± 0.62	Below LLoQ (5 fg/μL)
1 mg/h	46.48 ± 20.08	1.67 ± 0.72	Below LLoQ (5 fg/μL)
1.5 mg/h	62.92 ± 29.25	1.63 ± 0.57	0.0002 (0.00015–0.005) (*n* = 3)
2 mg/h	65.35 ± 27.59	2.00 ± 1.03	0.0006 (0.0002–0.002) (*n* = 4)
Mean AUC_0–24_ ± SD (mg*h/L)			
0.5 mg/h	585.91 ± 329.28	24.76 ± 11.90	n.a.
1 mg/h	932.57 ± 445.70	33.57 ± 15.15	n.a.
1.5 mg/h	1289.04 ± 576.65	32.71 ± 12.21	n.a.
2 mg/h	1335.87 ± 591.09	39.53 ± 23.07	n.a.
**60 mg nimodipine p.o. every 4 hours**			
Mean concentration of nimodipine ± SD (pg/μL)			Median (range)
60 mg 6×/day	11.81 ± 7.85	1.48 ± 0.98	0.00015 (0.0001–0.006) (*n* = 4)
Mean AUC_0–24_ ± SD (mg*h/L)			
60 mg p.o.	298.32 ± 206.52	34.80 ± 16.56	n.a.

AUC, Free area under the concentration time curve; CI, confidence interval; CSF, cerebrospinal fluid; ISF, interstitial fluid; LLoQ, lower limit of quantification; n.a., not available; SD, standard deviation.

Following oral administration of 60 mg nimodipine, mean drug concentration in plasma was 11.81 ± 7.85 pg/μL (**Table** **3**, **Figure** **1**). AUC_0–24_ for oral nimodipine in plasma was 298.32 (± 206.52) mg*h/L (**Table** **3**, **Figure** **2**).

#### CSF

The CSF concentration at intravenous infusion was about 10 times lower than in plasma (**Table** **3**, **Figure** **1**). Mean AUC_0–24_ in CSF was 24.76 (±11.9) mg*h/L, 33.57 (±15.15) mg*h/L, 32.71 (±12.21) mg*h/L, and 39.53 (±23.07) mg*h/L, respectively, for doses 0.5 mg/h–2 mg/h (**Figure** **2**). No significant increase in CSF concentration was observed between different intravenous dosings (*P* > 0.05, i.e., between 0.5 and 1 mg/h *P* = 0.14; between 1 and 1.5 mg/h *P* = 0.85, between 1.5 and 2 mg/h *P* = 0.33, between 0.5 and 1.5 *P* = 0.11, between 0.5 and 2 mg/h *P* = 0.1, between 1 and 1.5 mg/h *P* = 0.85, and between 1 and 2 mg/h *P* = 0.36; absolute values in **Table** **3**).

Penetration ratio from plasma to CSF was 3.8% (±1.5) after intravenous administration (mean of 0.5–2 mg/h) and 11.7% after oral administration (AUC_CSF_/AUC_plasma_).

Plasma concentration correlates significantly with CSF concentrations at 0.5 mg/h (*R* 0.7, *P* = 0.047) and 1 mg/h (*R* = 0.727, *P* = 0.017), but not at 1.5 mg/h (*R* = 0.51, *P* = 0.136) and 2 mg/h (*R* = 0.31, *P* = 0.383) infusion rates.

Following oral administration of 60 mg, mean nimodipine concentration in CSF was 1.48 ± 0.98 pg/μL. AUC_0–24_ for oral nimodipine in CSF was 34.80 (±16.56) mg*h/L (**Table** **3**, **Figure** **2**). Penetration ratio from plasma to CSF (AUC_CSF_/AUC_plasma_) was 17% (±11). Plasma concentrations did not correlate with CSF concentrations (*R* = 0.12, *P* = 0.78).

#### Cerebral ISF

**Figure 3:**
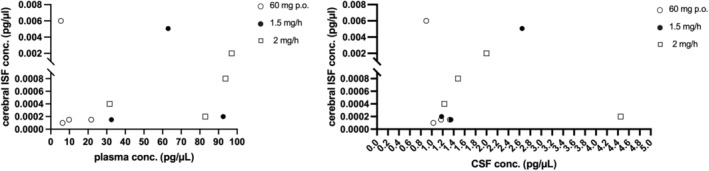
Nimodipine concentrations in cerebral interstitial fluid. Nimodipine concentration in cerebral ISF with corresponding plasma and CSF concentrations. conc., concentrations; CSF, cerebrospinal fluid; ISF, interstitial fluid.

Nimodipine was below the lower limit of quantification (LLoQ) (5 fg/μL) at an intravenous dose of 0.5 and 1 mg/h in all patients included (**Table** **3**). Nimodipine was detectable at a perfusion rate of 1.5 mg/h in three patients and at a perfusion rate of 2 mg/h in the same three patients and in an additional patient, in which data at 1.5 mg/h were unavailable due to microdialysis probe malfunction (**Table** **3**). A quantity of 60 mg of oral nimodipine administration resulted in detectable nimodipine concentrations in four out of nine patients (44.4%). Thereby, the patients exhibiting detectable nimodipine concentrations during oral administration were not identical to those who showed detectable concentrations at 1.5 mg/h and 2 mg/h continuous intravenous nimodipine administration. Only one patient had detectable nimodipine concentrations at 1.5 mg/h, 2 mg/h and at 60 mg oral nimodipine administration (**Figure** **3**).

#### Intravenous vs. oral nimodipine

Mean plasma concentration of nimodipine was the lowest following oral administration of 60 mg nimodipine and higher following intravenous infusion, showing a gradual increase from 0.5 mg/h infusion rate to 2 mg/h infusion rate (**Table** **3**). These differences in plasma between oral and intravenous administration were statistically significant for infusion rates of 0.5 mg/h (*P* = 0.008), 1 mg/h (*P* = 0.008), 1.5 mg/h (*P* = 0.008), and 2 mg/h (*P* = 0.008). The AUC_0–24_ was significantly higher in plasma after intravenous administration of nimodipine compared to oral administration (*P* = 0.008).

Mean nimodipine concentration in CSF was the lowest at 0.5mg/h of intravenous nimodipine infusion (1.28 ± 0.62 pg/μL). During different doses of intravenous infusion, a small dose‐dependent increase in nimodipine concentration in CSF was observed from 1.28 ± 0.62 pg/μL at 0.5 mg/h infusion rate to 2 ± 1.03 pg/μL at 2 mg/h (**Table** **3**). These differences in CSF concentration between oral and intravenous administration were statistically not significant for infusion rates of 0.5 mg/h (*P* = 0.94), 1 mg/h (*P* = 0.21), 1.5 mg/h (*P* = 0.31), and 2 mg/h (*P* = 0.17).

The AUC_0–24_ was significantly higher in CSF after intravenous administration of nimodipine at doses ≥1 mg/h compared to oral administration (between oral and 0.5 mg/h *P* = 0.25, between oral and 1 mg/h *P* = 0.012, between oral and 1.5 mg/h *P* = 0.05, and between oral and 2 mg/h *P* = 0.05).

## DISCUSSION

In this prospective study, nimodipine was measured for the first time systematically in plasma, CSF, and brain parenchyma concomitantly. We were able to demonstrate that nimodipine was found at consistently higher concentrations in CSF at intravenous nimodipine infusions of ≥1 mg/h (*P* < 0.05) compared to oral intake of 60 mg every 4 hours. In a prior study,^11^ aSAH patients with a favorable long‐term functional outcome (mRS 0–1) exhibited significantly higher CSF nimodipine concentrations compared to those with poor outcome (mRS ≥2). The increased concentration in CSF following intravenous infusion may contribute to a more favorable functional outcome, as higher nimodipine concentrations can effectively reach the smallest blood vessels by diffusing into the Virchow‐Robin space, thereby exerting its enhanced vasodilatory effects. This suggests that CSF concentrations of nimodipine may play a more crucial role in improving outcomes than cerebral ISF concentrations.^11^ Additionally, the substantial interindividual variability in cerebral exposure poses a challenge to the proposed neuroprotective effects of nimodipine.

In previous studies, the mean CSF concentrations demonstrated concentration of nimodipine of about 0.3 pg/μL,^17^ whereas in our cohort, we observed concentrations of 1.28–2.0 pg/μL.

Interestingly, although the total amount of nimodipine reaching CSF was higher at intravenous infusion at >1 mg/h, the ratio of drug concentration in the CSF following oral administration was higher than that at intravenous infusion, despite the considerably lower total plasma concentration at oral administration. Oral administration in this study was generally later after the initial bleeding event than intravenous administration. Consequently, the degree of brain edema and inflammation changed over time,^18^ affecting blood–brain barrier permeability and leading to increased relative brain penetration. As a cross‐over study is unfeasible in this critically ill patient cohort, an analysis of the patients' hemodynamic parameters over time and intracerebral metabolites may help to answer this question in the future.

The pharmacokinetics of nimodipine in plasma and CSF have been studied in healthy volunteers and patients with subarachnoid hemorrhage,^17^ showing 36–72 pg/μL^17^ and 27–53 pg/μL,^3^ respectively, in plasma after intravenous infusion, which is similar to our results. On the contrary, they observed higher plasma concentrations after oral administration of 60 mg nimodipine (17–42 pg/μL and up to 31 pg/μL after one hour, respectively^3^, ^17^) compared to 11.81 ± 7.85 pg/μL at steady state in our cohort. As a consequence, the relative plasma bioavailability in our cohort was lower than the previously reported 4.8–8.8%.^16^ Still, it has to be considered that we calculated the steady‐state concentration, compared to peak concentration after one hour in the literature.^17^ Moreover, while Rämsch et al.^17^ calculated pharmacokinetic parameters in a well‐defined study cohort and healthy volunteers, our data reflect real‐world data, which may explain the even lower bioavailability in our cohort. We obtained samples from severely ill patients with a mean Hunt&Hess of 4 ± 1, who were deeply sedated, with close intracranial pressure monitoring, a 30° elevation of the upper body and restriction of moving the body. This suggests that gastrointestinal function was probably reduced in our cohort, also explaining the reduced relative plasma bioavailability.

Animal models have demonstrated that nimodipine can effectively traverse the blood–brain barrier.^19^, ^20^ To the best of our knowledge, there is only one study measuring nimodipine in human brain parenchyma using cerebral microdialysis after oral administration of nimodipine in aSAH so far.^21^ However, they were not able to measure nimodipine concentration in CSF and cerebral ISF in 94% of all cases. The discrepancy to our results may be attributed to the considerably higher LLoQ (0.5 ng/ml) in this previous study, which markedly exceeds the limit employed in the present investigation.^21^ By using a much more sensitive LLoQ (5 fg/μL) and carefully protecting the samples from light, we successfully quantified nimodipine in CSF in all samples. Additionally, nimodipine was detected in ISF in 40% of the patients examined. However, the recovery rate of nimodipine in cerebral microdialysis exhibited considerable variability in *in vitro* experiments. Therefore, nimodipine, as a substance, may not be ideal for precise quantitative measurements of cerebral ISF concentrations using microdialysis.

Furthermore, nimodipine is a substrate of P‐gp,^14^, ^15^ which may result in nonlinearity of drug concentrations between CSF and cerebral ISF as well.^22^ This is reflected in the significant difference between CSF and cerebral ISF concentrations and the CSF/plasma ratio in our study. Nevertheless, these findings suggest that at least a minor fraction of nimodipine traverses the blood–brain barrier.

Plasma concentrations showed a gradual increase in a dose‐dependent manner. Importantly, plasma concentration frequently correlates with clinically relevant hypotension that may necessitate the administration of vasopressors.^13^ In a prior investigation, we demonstrated that the oral administration of 60 mg nimodipine every 4 hours resulted in a reduced arterial blood pressure, cerebral perfusion pressure (CPP), and brain tissue oxygen (ptiO_2_).^13^ However, circulatory support with norepinephrine mitigated these side effects, preserving cerebral metabolism unaffected. It remains unclear whether a similar effect can be observed with intravenous administration. Interestingly, there is only a minimal difference in CSF concentrations among 1, 1.5, and 2 mg/h of nimodipine infusions. The peripheral vasodilatory effect of nimodipine, which may result in hypotension, is accompanied by a reduction in cardiac output and changes in regional blood flow. Consequently, the extent of drug distribution within organs becomes less predictable. This is of particular interest in aSAH, as alterations in cerebral blood flow and cerebral blood volume are associated with changes in blood–brain barrier permeability.^23^ Hence, fluctuations in hemodynamics also impact cerebral blood flow and volume and may explain the non‐linearity in CSF pharmacokinetics observed at distinct intravenous doses and oral dose in this cohort.

Therefore, decreasing the dose from 2 to 1.5 mg/h (or even 1 mg/h) might reduce hemodynamic side effects without impairing cerebral nimodipine exposure (at least in our study, nimodipine was undetectable in cerebral ISF at 1 mg/h, preferring 1.5 or 2 mg/h to 1 mg/h).

Despite no significant difference in the absolute concentration of nimodipine in the CSF following the administration of 60 mg orally and a continuous intravenous infusion at a rate of 2 mg/h, the intravenous administration of nimodipine allows for a precise control of plasma levels, thereby avoiding peaks and troughs that may result from oral administration and cause fluctuations in effect.^23^ The continuous infusion may result in a more consistent therapeutic effect, as indicated by the significantly higher AUC in our results; however absolute concentrations were not significant. Intravenous infusion may lead to more consistent cerebral blood flow and perfusion, which could potentially reduce the incidence of severe vasospasm. Additionally, patients with SAH are critically ill, which can affect the absorption of oral nimodipine and the first‐pass mechanism in the liver,^24^ leading to unpredictable plasma and CSF concentrations. However, further prospective studies are necessary to confirm these findings.

The following limitations of this study have to be considered: (1) First of all, the small sample size. While the number of patients included is adequate for pharmacokinetic analysis, it is important to note that no definitive conclusions, particularly regarding outcome measurements, can be extrapolated from this population. (2) Nimodipine was initially administered intravenously to all patients, with a subsequent switch to oral administration after 10–14 days. As a result, intravenous concentrations were measured during the critical phase of aSAH, and oral concentrations were assessed thereafter, potentially influencing results and outcome. To eliminate this confounder, a cross‐over study would have been required; however, conducting such a study would have been unethical in this vulnerable population.

## CONCLUSION

In aSAH patients, significantly higher concentrations of nimodipine can be achieved in both plasma and CSF during intravenous compared to oral administration. Conversely, in cerebral ISF, only low amounts of nimodipine are found after both routes, with detection limited to less than half of the patients. These findings strongly suggest that nimodipine primarily exerts its beneficial effect on functional outcome by impeding cerebral ischemia through vasodilation via the blood–CSF barrier rather than neuroprotection via the blood–brain barrier. Moreover, the significantly higher AUC_0–24_ of nimodipine in plasma, as well as highest CSF concentration following 2 mg/h intravenous infusion, suggests that this route of administration may be more clinically effective in terms of outcome than oral administration, as it ensures more consistent nimodipine exposure.

Our results support the use of intravenous nimodipine instead of oral nimodipine in patients with aneurysmal subarachnoid hemorrhage. Whether the significantly higher CSF nimodipine concentrations at 2 mg/h infusion translate into significantly less delayed cerebral ischemia and better neurological outcome needs to be investigated in a prospective randomized trial.

## FUNDING

This study was funded by the Austrian Science Fund (FWF) (KLI 947‐B).

## CONFLICT OF INTEREST

The authors declare no competing interests for this work.

## AUTHOR CONTRIBUTIONS

M.M.M. and A.H. wrote the manuscript; M.M.M., K.R., M.Z., and A.H. designed the research; M.M.M., K.R., D.H., L.G., W.P., J.H., A.R., and M.Z. performed the research; M.M.M., A.T., M.Z., and A.H. analyzed the data.

## CONSENT TO PARTICIPATE

Patients meeting study criteria were initially unable to provide written consent due to sedation and mechanical ventilation. Upon regaining consciousness, patients were informed about the study and retrospective permission was obtained in case the patients regained consciousness.

## Data Availability

The datasets used and/or analyzed during the current study are available from the corresponding author on reasonable request.
